# Draft Genome Sequence of Fish Pathogenic *Vibrio vulnificus* Biotype 2

**DOI:** 10.1128/genomeA.01224-14

**Published:** 2014-11-26

**Authors:** Yael Koton, Saleh Eghbaria, Michal Gordon, Vered Chalifa-Caspi, Naiel Bisharat

**Affiliations:** a Department of Medicine D, Emek Medical Center, Afula, Israel; b Bruce Rappaport Faculty of Medicine, Technion–Israel Institute of Technology, Haifa, Israel; c Bioinformatics Core Facility, National Institute for Biotechnology in the Negev, Ben-Gurion University of the Negev, Be’er-Sheva, Israel

## Abstract

*Vibrio vulnificus* is a marine pathogen capable of causing severe soft tissue infections and septicemia in humans. *V. vulnificus* biotype 2 is the etiological agent of fish vibriosis. We describe here the first draft genome sequence of *V. vulnificus* biotype 2, strain ES-7601, isolated from an infected eel in Japan.

## GENOME ANNOUNCEMENT

*Vibrio vulnificus* is a bacterial pathogen that inhabits brackish waters worldwide. *V. vulnificus* strains are divided into three biotypes based on their biochemical characteristics. Biotype 1 is the most abundant and is the primary cause of human disease worldwide (1). Biotype 2 primarily affects fish and is considered to be the etiological pathogen of fish vibriosis (hemorrhagic septicemia) (2). Biotype 3 has been described only in Israel and affects humans only (3). Biotype 2 strains are subdivided into at least three different O-antigenic serovars (A, E, and I) (4, 5), serovar E being the one associated with human vibriosis (6). It has been suggested that biotype 2 virulence to fish is mediated by a 68-kb plasmid (7, 8).

Here, we describe the draft genome of strain ES-7601 (reference bacterial strain ATCC 33147/CECT 897/ NCIMB 2136), a *V. vulnificus* biotype 2 (serovar E), isolated from an infected eel in Japan in 1979. A total of 74 ng/µL genomic DNA was extracted using a commercial kit (Qiagen DNeasy kit). Adaptors were added to each library during preparation according to the TruSeq protocol (Illumina) to produce multiplexed paired-end libraries. The sample was run on a sequencer (Illumina MiSeq) at the Technion Genome Center, Haifa, Israel, generating 6,714,846 paired-end-reads. Sequence read data were mapped to 2 reference genomes (CMCP6 and YJ016) with an average coverage of 250×. The genome was *de novo* assembled with SPAdes (9) and RAST (Rapid Annotation using Subsystem Technology) (10) was used for gene annotation.

The assembled genome consisted of 281 contigs totaling 5,066,572 bp in length, the largest of which was 254,323 bp with an *N*_50_ length of 68,299 bp. A total of 4,411 coding sequences were identified with 172 pseudogenes, 9 rRNAs, 99 tRNAs, and 1 non-coding RNA (ncRNA). A BLAST analysis was performed to identify sequences in the draft genome sharing sequence similarity to the plasmids that have been described in *V. vulnificus* biotype 2. The genome sequence of strain ES-7601 showed ≥98% nucleotide sequence similarity to *V. vulnificus* plasmid pC4602-1 (56 kb).

### Nucleotide sequence accession numbers.

This whole-genome shotgun project has been deposited at DDBJ/EMBL/GenBank under the accession no. JRQR00000000. The version described in this paper is version JRQR01000000.
